# The impact of perceived ethnic discrimination on mental health depends on transcultural identity: evidence for a moderator effect

**DOI:** 10.1186/s40359-015-0088-x

**Published:** 2015-08-29

**Authors:** Miriam A. Knauss, Kristina Günther, Sophie Belardi, Pauline Morley, Ulrike von Lersner

**Affiliations:** Freie Universität Berlin, Department of Educational Science and Psychology, Habelschwerdter Allee 45, 14195 Berlin, Germany; Department of Psychology, Humboldt-Universität zu Berlin, Rudower Chaussee 18, 12489 Berlin, Germany

## Abstract

**Background:**

While ethnic discrimination emphasizes boundaries between different cultures, the concept of transculturality focuses on the fact that cultures can merge and that individuals integrate novel cultural elements into their identity. This is an exploratory study that investigates the interplay between perceived ethnic discrimination, psychological adjustment and transcultural identity.

**Methods:**

Structured interviews were conducted using a sample of 46 adolescents with a Turkish migratory background and 45 adolescents who were native born Germans.

**Results:**

Correlational and multiple regression analyses revealed that perceived discrimination was clearly associated with a poorer psychological adjustment among adolescents with a migratory background. Transcultural identity moderated this relationship. That is, adolescents who showed higher levels of transcultural identity displayed a better psychological adjustment when compared to adolescents who showed lower levels of transcultural identity—provided that they did not feel discriminated against. This is congruent with the idea that transcultural identity can involve considerable benefits for personality. However, when adolescents perceived higher rates of discrimination, higher levels of transcultural identity came attached to a poorer psychological adjustment.

**Conclusions:**

The findings suggest that perceived discrimination has negative effects on the well-being of immigrant adolescents—particularly for those who describe their identity as transcultural. The findings are discussed considering specific characteristics of transcultural identity, and how they stand in opposition to discrimination.

## Background

International studies on migration and mental health have highlighted the role of perceived ethnic discrimination as an important stressor for persons with a migratory background. Perceived discrimination refers to the belief that one has been disadvantaged because of one’s origin (Mesch et al. 2008). Due to its subjective nature, perceived discrimination does not only reflect “objective” discriminatory occurrences but may also involve subtle or ambiguous experiences that outside observers might not attribute to discrimination (Clark et al. 1999). A number of comprehensive literature reviews find substantial evidence that persons who perceive discrimination upon themselves have more physical health problems, such as hypertension and heart disease (Williams & Mohammed 2009). More so, perceived discrimination has been found to be associated with mental health problems, such as depressive symptoms and anxiety (Paradies 2006; Williams et al. 2003). This is evident in adults (Chou et al. 2012; Lee & Ahn 2012; Pascoe & Richman 2009) as well as in adolescents (Brody et al. 2006; Galliher et al. 2011; Seaton et al. 2010).

However, the relationship between perceived discrimination and mental health varies between regions of investigation (Lee & Ahn 2012). Besides methodological differences between studies, this could be due to different living conditions and political situations (e.g., immigrant rates and immigrants’ rights). In the case of Germany, 41 percent of immigrants feel confronted with discrimination (Federal Statistical Office of Germany 2012), which points to the relevance of this topic. Compared to other ethnic minority groups, people with a Turkish migratory background are the group most affected by experiences of discrimination.

The *migration-stress-paradigm* (Schepker & Toker 2009) hypothesizes that—due to experiences of discrimination and other acculturative experiences—immigrant adolescents exhibit a poorer psychological adjustment than their peers without a migratory background. However, recent studies did not provide evidence for this assumption (Stevens & Vollebergh 2008; von Lersner et al., unpublished observations). Antonovsky (1987) claimed that the ability to overcome negative life events or stressors is more important for mental health than the absence of negative experiences per se. This suggests that risk and resiliency factors should be taken into account when studying psychological adjustment and its relation to perceived discrimination.

### Cultural identity and perceived discrimination

When members of different cultures encounter each other, this entails mutual adaptation processes that further influence cultural identity (Berry 1990). In terms of these *acculturation processes*, individuals negotiate to which degree they identify with the society of their origin or seek involvement with the majority society (Berry et al. 2006; Phinney 1990). Forming a coherent identity is a central task of individual development, especially during adolescence (Erikson 1963). The sense of belonging to the society of one’s origin defines *ethnic identity* (Phinney 1990). Interestingly, ethnic identity serves as a protective factor in the context of perceived discrimination and psychological adjustment (Galliher et al. 2011; Mossakowski 2003; Vera et al. 2011; Wong et al. 2003). That is, persons who feel discriminated against and at the same time identify strongly with the society of their origin would have a better psychological adjustment when compared to persons who feel discriminated against and identify less with their society of origin. Furthermore, perceived discrimination is also directly linked to ethnic identity (Phinney 1990): Persons who experience more discrimination identify more strongly with the society of their origin and vice versa. Sellers et al. (2006) further specified that this holds especially for the facet *public regard* (e.g. “Others respect Black people”) of ethnic identity but not necessarily to other facets such as *private regard* (e.g. “I feel good about Black people”).

When an individual incorporates the majority society’s culture as well as the culture of the ancestry society into a cohesive sense of identity, this is referred to as *bicultural identity* (Phinney 1990). Benet-Martinez and Haritatos (2005) extended and measured the concept of bicultural identity with the Bicultural Identity Integration (BII) scale. It includes two factors, *conflict* and *distance*, that indicate the degree to which an individual confronted with two cultures perceives them as overlapping and integrated versus opposed and difficult to integrate. The factor *conflict* refers to affective aspects of biculturality whereas the factor *distance* includes learning and performance aspects of acculturation (Benet-Martinez & Haritatos 2005). Individuals experiencing high cultural conflict feel ambiguous with regard to their cultural affiliation. Individuals with high cultural distance are likely to state that they keep cultures separate. Low BII can be a source of internal conflict, whereas individuals that score high on BII often feel as being part of a new mixed culture with dissolving cultural boundaries (Benet-Martinez & Haritatos 2005). BII is inversely associated with perceived discrimination: Individuals who perceive discrimination appear to have more difficulty in incorporating the cultures of both mentioned societies into the self (Benet-Martinez & Haritatos 2005).

The findings on ethnic and bicultural identity suggest that cultural identity is closely connected to the perception of discrimination and might even determine how people cope with experiences of discrimination. These findings depend substantially on the operationalization of cultural identity. Therefore, one aim of this study is to incorporate recent developments in the conceptualization of cultural identity.

### Transcultural identity

Due to the increasing mobility in the contemporary world, new forms of interaction between cultures emerge. Local cultures are influenced by migration and globalization, e.g. by global media, free market economics and democratic institutions, which in turn affects traditional cultural practices and beliefs (Arnett 2002). According to Welsch (2010), these changes have to be integrated appropriately in current concepts of culture: the assumption that immigrants generally assimilate to majority society and lose the cultural traits of their society of origin does not hold (Suárez-Orozco & Suárez-Orozco 2009). Rather than co-existing separately from each other, different cultures can merge and cultural boundaries can become blurred or even disappear (Welsch 2010). This merging of cultures is not only reflected at the societal level, but also in identity building at the individual level: for example, one may have to incorporate diverse elements of the ancestry culture, the local culture, and the global culture into one’s identity (Welsch 2010). According to Suárez-Orozco and Suárez-Orozco (2009), there are three different styles of identity adolescents with a migratory background are likely to develop: *Ethnic flight* (abandoning the society of their origin and adapting to the majority society), *adversarial identities* (constructing identity in opposition to the majority society and its institutions) and *transcultural identities.* The concept of transcultural identity, like bicultural identity, centers on the integration of cultures but extends its scope in several aspects (Günther 2012, unpublished observations). Individuals who have developed a transcultural identity incorporate different cultural influences and transform elements of different cultures into new forms and practices (Pieterse 1994; Rowe & Schelling 1991), or into "a new cultural space” (Welsch 1995). This new cultural space originates in the fusion and entanglement of cultures and thereby transcends classical national cultural frames (Welsch 1995, 2010). The heterogeneity that results appears in different domains of everyday life like music, food, series and films on television, sports clubs, language, religion, annual festivals, clothing style. The concept of transcultural identity is in some ways similar to the concept of BII. In both concepts, the emergence of a new cultural space is discussed. Whereas biculturals only experience this when they perceive little cultural distance and low conflict, in the transcultural approach the emergence of a cultural space is seen as a consequence of converging cultures (Günther 2012, unpublished observations). Moreover, in a transcultural identity, an indefinite amount of cultural influences can be combined. Hermans and Kempen (1998) described the phenomenon of transcultural identity under the name of *hybrid identity.* Arnett (2002) stated that changes due to globalization “may lead less to a bicultural identity than to a hybrid identity”. Persons without a migratory background may also have a transcultural identity—and not everybody who is confronted with at least two cultures is automatically supposed to have a transcultural identity (Welsch 2010). Thus, biculturals do not necessarily have a transcultural identity.

According to Suárez-Orozco and Qin (2006), an important factor that affects identity formation is *social mirroring*. A social mirror reflects the image of an ethnic group held by the majority society, e.g. by teachers, police officers, in the media etc. How is identity formation affected by perceived discrimination? One possibility is that people who experience discrimination are less likely to develop a transcultural identity. Since transcultural identity is viewed as the most adaptive identity style for adolescents with a migratory background (Suárez-Orozco & Suárez-Orozco 2009), it is of interest whether transcultural identity also serves as a resiliency factor, with the potential to buffer the negative effects of perceived discrimination.

### The current study

To the knowledge of the authors, there have been no attempts to measure transcultural identity empirically thus far. The present study represented a pioneering effort to quantify transcultural identity and to explore its relation to perceived discrimination and basic indicators of psychological adjustment, such as depressive symptoms, anxiety, aggressive behavior or anger control problems. Specifically, we investigated whether perceived discrimination was associated with a poorer psychological adjustment among adolescents with a migratory background, and if there were general differences regarding psychological adjustment between adolescents with and without a migratory background. With regard to transcultural identity we explored whether adolescents with discrimination experiences were more or less likely to have a transcultural identity. Transcultural identity was assessed as a continuum with higher scores indicating high degrees of incorporation of various elements of different cultures into the self. Importantly, this research also investigated if transcultural identity served as a risk or resiliency factor for adolescents who perceived discrimination.

## Method

### Procedure

Participants were recruited from schools and youth centers in Berlin. To increase generalizability, the study only included schools and youth centers located in three boroughs of Berlin whose immigrant rates are similar to Berlin’s overall immigrant rate. According to the Bureau of Statistics Berlin Brandenburg (Bureau of Statistics Berlin Brandenburg 2011) 26.5 % of the inhabitants in Berlin have a migratory background. The schools’ immigrant rates did not differ significantly from each other, or from the overall immigrant rate of Berlin. After taking into account gender and immigration status, participants were selected randomly. We conducted interviews in an individual setting at the schools and youth centers. All interviewers were trained in at least five sessions with pilot participants (students recruited from Humboldt-Universität zu Berlin) and individual feedback given by a professional supervisor. The items were read out loud and the responses for each item were given on Likert-type scales which were visualized for the participants on a sheet of paper. Each interview lasted 60–90 min and participants received a gift card as a reward after the interview.

### Participants

The sample included *N =* 91 adolescents aged 14 to 17 years (*M* = 15.0, *SD* = .84) with a proportion of 47 (51.6%) female and 44 (48.4%) male. Forty-six (50.5%) of the participants had a Turkish migratory background, the remaining 45 participants (49.5%) were native-born Germans. Adolescents of the German sample who were born in another country, or had a parent or grand-parent that was born in another country, were not included in the sample. As people of Turkish origin represent the largest group of immigrants in Germany, the current study focused on adolescents with a Turkish migratory background. A Turkish migratory background implied that the participant or at least one of his or her parents or grandparents was born in Turkey (Siefen & Boos-Nünning 2005). Except for two participants, all subjects of the subsample with a migratory background were born in Germany. Young people with a Turkish migratory background in Germany are predominantly second-generation immigrants, and the majority of them possess German citizenship (German Federal Statistical Office 2012). In Berlin, around half of the young people between 15 and 18 years have a migratory background and in some boroughs the proportion is around two thirds (Amt für Statistik Berlin Brandenburg [Bureau of Statistics Berlin Brandenburg] 2011).

The ethical approval for the conduction of the study was obtained from the Senate Office of Science Berlin. Informed written consent was obtained before the experiment from the school directors or coordinators of the youth centers, respectively. As adolescents in Germany have the right to self-disclosure from the age of 14, we did not obtain consent from the parents of the participants. For the students participation in the study was voluntary. The response rate was good, except for two, all adolescents approached for the study agreed to participate.

### Measures

The investigation was conducted in an interview format. The demographic variables and instruments included in the interview are described below.

#### Demographic variables

We examined and controlled for the demographic variables age, gender, borough, social desirability, school type, and marital status of the parents. For reasons of data protection, we did not have the permission to ask the participants about their social status. As families with low income are exempted from co-payments for school books in Germany, we assessed social status approximately with the variable *exemption from the co-payment of learning and teaching aids*. This indicator of social status was also included in the analyses. 37 % (*n* = 17) of the participants from Turkish families stated to be exempted from the co-payment of learning and teaching aids and 15.6 % (*n* = 7) of the participants from German families did. The higher rate stated by adolescents with a migratory background suggests a lower average social status in this group.

#### Psychological adjustment

Adolescents’ psychological adjustment was assessed using the *Screening of psychological disorders in adolescence* (SPS-J) by Hampel and Petermann (2006), the German version of the *Reynolds’ Adolescent Adjustment Screening Inventory* (Reynolds 2001). It is a brief self-report measure investigating symptoms of externalizing and internalizing behavior in the past six months. It consists of 32 items and includes four subscales (*anger control problems* and *aggressive antisocial behavior* as well as *depressive symptoms* and *problems of self-esteem*). The overall value of these four subscales will be labeled as *psychological distress* in the following. The items were rated on a three-point Likert-type scale ranging from 1 *(hardly ever)* to 3 *(almost all of the time)*. Additionally, the frequency of somatic symptoms in the recent weeks was assessed using a questionnaire by Grob et al. (1991) including a four-point Likert scale from 1 (*never*) to 4 (*very often*). Internal consistencies were satisfying, Cronbach’s α = .82 and α = .68 for the overall value of *psychological distress* and for *somatic symptoms*, respectively.

#### Perceived discrimination

The questions concerning perceived discrimination were included in the interviews with participants with a migratory background only. The items were derived from Skrobanek’s (2007) questionnaire that distinguishes between two dimensions of discrimination: *perceived personal* and *perceived group discrimination*. In the original questionnaire, each dimension is assessed by four items referring to the perception of disadvantages compared to persons with a German ancestry in different areas of public life (*school*, *work*, *leisure time*). In order to cover a greater range of different areas of life, two further questions were added to the group discrimination scale (*law*, *housing*) and nine further questions to the personal discrimination scale—asking about further areas of public life (*public transport*, *police*, *neighborhood*, *shops*) and different groups of persons (*teachers*, *adults outside of school*, *classmates*, *adolescents outside of school*). The latter items were derived from Berry et al. (2006). According to an exploratory factor analysis, the supplementary items were assignable to Skrobanek’s (2007) original factors. Both scales had a four-point response format with a range from 1 (*don’t agree at all*) to 4 (*agree very strongly*) for *perceived group discrimination* and from 1 (*not at all*) to 4 (*very strong*) for *perceived personal discrimination*. Where not applicable (e.g., when a participant never went to the youth club, thus rendering items that referred to the youth club redundant) values were estimated by the mean (sumscore of a person divided by the number of responded items). Reliabilities turned out satisfactory with Cronbach’s α = .86 and α = .79 for personal discrimination and group discrimination, respectively. In addition, we coded whether female participants wore a headscarf or not. In our sample, this was only the case for two participants.

#### Transcultural identity

The items assessing transcultural identity were developed by the research group based on the concept of transculturality as described by Welsch (1995) and the *BII Scale* by Benet-Martinez and Haritatos (2005). Accordingly, we measured the emergence of a new cultural space, neglect of cultural boundaries, and the involvement in more than one cultural context. A sample item is: *I am a part of a new culture, because inside of me, influences of different cultures have been mixed*. The scale had four response points from 1 (*don’t agree at all*) to 4 (*agree very strongly*). Internal consistencies were acceptable, Cronbach’s α = .69. A further evaluation of the transcultural identity scale is provided in the Appendix section.

#### Social desirability

Higher scores in social desirability come along with fewer reports on depressive and anxiety symptoms (Logan et al. 2008). Thus, we controlled for social desirability using the short form of the *Social Desirability Scale-17* by Stöber (1999). Internal consistency is reported to range from .72 to .75.

### Data analyses

As a first step, we tested data for univariate and multivariate outliers according to Tabachnick and Fidell (2007) using box-plot graphs as well as z-values and Mahalanobis distance. Concerning the variable *perceived discrimination*, one outlier was identified. This outlier was within the valid range of values, and could not be traced back to an input data error, a misunderstanding, or a boycott (Eid et al. 2010). Therefore the case was not excluded from the data set.

The means of psychological adjustment indicators for the participants with and without a migratory background were compared using *t*-tests. The relationship between perceived discrimination and psychological adjustment was analyzed with correlational and multiple regression analyses. Perceived discrimination was entered each time as a last step in the analysis.

We explored whether the association of perceived discrimination and psychological adjustment depended on a third variable—transcultural identity—in a moderator analysis. Analogous to the procedure described by Eid et al. (2010) the variables *perceived discrimination* and *transcultural identity* as well as their product were entered into the regression analysis. In order to avoid multicollinearity, the variables *perceived discrimination* and *transcultural identity* were centered before entering into the regression analysis.

Errors in the regression analysis were not uncorrelated. This can probably be traced back to the nested structure of the data. Participants were situated within different boroughs and schools, which represent different aggregate units. In order to control for *borough*, this variable was dummy coded and entered into the regression analysis according to the recommendation by Eid et al. (2010). The variable *school* was not entered in the regression analysis because it would have exceeded the recommended maximum number of predictors.

## Results

Table 1 provides correlations, ranges, means and standard deviations of all variables for the participants with a migratory background. Means and standard deviations for the participants without a migratory background are displayed at the bottom of the table.

**Table 1:** Correlations, ranges, means and standard deviations of the study variables for the sample with migratory background. means and standard deviations for the sample without migratory background are presented in the bottom line

Variable	1	2	3	4	5	6	7	8	9	10
1 Personal discrimination	-									
2 Group discrimination	.61**	-								
3 Overall discrimination	.95**	.83**	-							
4 Depressive symptoms	.42**	.29*	.41**	-						
5 Problems of self-esteem	.22	.25	.26	.21	-					
6 Anger control problems	.37*	.21	.34*	.10	.56**	-				
7 Aggressive behavior	.58**	.41**	.57**	.19	.37*	.67**	-			
8 Overall psych. distress	.59**	.42**	.58**	.62**	.64**	.78**	.78**	-		
9 Somatic symptoms	.70**	.47**	.68**	.45**	.26	.31*	.47**	.55**	-	
10 Transcultural identity	−.06	.02	−.04	.01	−.00	−.02	.02	.00	−.02	-
Variable range	1–4	1–4	1–4	0–20	0–12	0–16	0–16	0–64	1–4	1–4
Cut-off values				10	9	8	7	27		
*M (SD)*	1.56 (0.45)	2.14 (0.55)	1.75 (0.44)	7.85 (3.85)	2.83 (1.62)	4.52 (3.00)	3.20 (2.93)	18.39 (8.06)	1.80 (0.52)	1.93 (0.54)
*M (SD)* sample without migr. backgr.		-	-	6.24 (3.40)	2.49 (1.85)	4.42 (1.94)	3.91 (2.29)	17.07 (6.73)	1.75 (0.49)	2.07 (0.56)

*n* = 46 for the sample with a migratory background. *n* = 45 for the sample without a migratory background

*p < .05 **p < .01

### Perceived discrimination

Sixty-three percent (*n* = 29) of the participants with a migratory background reported having experienced strong or very strong personal discrimination in at least one of the areas of public life covered in the interview. Concerning the variable *perceived group discrimination*, 69.6% (*n* = 32) agreed in at least one item that persons with a Turkish migratory background are disadvantaged compared to persons who had a German ancestry. A Wilcoxon test showed that perceived group discrimination (*M* = 2.14, SD = 0.55) was significantly higher than personal discrimination (*M* = 1.56, SD = 0.45), *z* = 5.59, *p* < .01, *ω* = .62. Highest scores of personal discrimination were reported to be emanating from teachers (*M* = 1.89, SD = 0.85) and adults outside of school (*M* = 1.83, SD = 0.77) as well as on the streets or in public transport (*M* = 1.85, SD = 0.95). Lowest scores of discrimination were reported to emanate from classmates (*M* = 1.22; SD = 0.47).

### Psychological adjustment

The psychological adjustment scores in the SPS-J questionnaire were comparable to those of the standard sample indicated in the test manual. Regarding overall psychological distress, participants with a migratory background (*M* = 18.39, SD = 8.06) and native German participants (*M* = 17.07, SD = 6.73) did not differ significantly, *t*(89) = .85, *p* > .05, *ω* = .01. However, participants from Turkish families showed higher scores in the subscale *depressive symptoms*, *t*(89) = 2.10, *p* < .05, *ω* = .05. When controlling for social status, this difference disappeared, *F*(1, 71) = 1.43, *p* > .05, *ω* = .02. It has to be noted that the variable *social status* exhibited 18.7 % (*n* = 17) of missing values. Hence, we omitted cases with missings concerning *social status* when controlling for this variable. The psychological adjustment of participants with missing data regarding their social status did not differ systematically from that of other participants, *t*(89) = .31, *p* > .05, *ω* = .00.

Regarding the full sample, girls reported slightly more depressive symptoms than boys (*t*(89) = −2.42, *p* < .05, *ω* = .06) whereas boys reported more aggressive antisocial behavior (*t*(89) = 2.03, *p* < .05, *ω* = .04). However, within the group holding a migratory background, no gender differences were observed (*t*(44) = −1.83, *p* > .05, *ω* = .07 and *t*(44) = .61, *p* > .05, *ω* = .01 for depressive symptoms and aggressive antisocial behavior, respectively).

### Perceived discrimination and psychological adjustment

Perceived discrimination was associated with a poorer psychological adjustment (see Table 1 for statistics). Thus, adolescents who perceived personal discrimination or group discrimination showed higher levels of depressive symptoms, aggressive antisocial behavior, anger control problems, overall psychological distress, as well as higher levels of somatic symptoms.

The results of the regression analysis concerning perceived personal and group discrimination are displayed in Table 2. Multiple regression analysis controlling for *age*, *gender*, *borough*, *social status*, *marital status of the parents*, *social desirability*, and *school type* also revealed that aggressive antisocial behavior, anger control problems, overall psychological distress, as well as somatic symptoms could be predicted by overall perceived discrimination and perceived personal discrimination. Depressive symptoms were more reliably predicted by demographic variables than by perceived discrimination.

**Table 2:** Stepwise multiple regression analyses predicting outcomes of psychological adjustment by perceived discrimination

Outcome	Predictor	*B*	*SE B*	*ß*	*R* ^*2*^	Δ*R* ^*2*^
Aggressive antisocial behavior						
Step 1	Constant	19.32	2.74		.21	
	Control variables^a^	−2.58	.85	.46**		
Step 2	Constant	13.47	2.96		.41	.20**
	Control variables^a^	−2.38	.75	.43**		
	Personal discr.	3.41	1.02	.45**		
Anger control problems						
Step 1	Constant	24.00	3.02		.30	
	Control variables^a^	−3.54	.94	.55**		
Step 2	Constant	17.91	3.32		.46**	.16**
	Control variables^a^	−3.33	.84	.51**		
	Personal discr.	3.55	1.14	.40**		
Overall psychological distress						
Step 1	Constant	70.27	8.15		.15	
	Control variables^a^	−6.17	2.53	−.39*		
Step 2	Constant	50.67	8.40		.42**	.28**
	Control variables^a^	−5.49	2.12	−.34*		
	Personal discr.	11.44	2.88	.53**		
Somatic symptoms						
Step 1	Constant	1.34	.19		.13	
	Control variables^a^	.07	.03	.36*		
Step 2	Constant	.26	.30		.43**	.30**
	Control variables^a^	.07	.03	.35*		
	Personal discr.	.72	.17	.55**		
Step 1	Constant	1.34	.19		.13	
	Control variables^a^	.07	.03	.36*		
Step 2	Constant	.56	.37		.26	.13*
	Control variables^a^	.07	.03	.35*		
	Group discrimination	.38	.16	.37*		

*n* = 36. *p < .05 **p < .01

^a^Control variables including age, gender, borough, social status, social desirability and school type

When controlling for demographic variables, perceived group discrimination predicted only somatic symptoms—not the other outcomes of psychological adjustment. Hence, personally experienced discrimination in particular plays a role for psychological adjustment.

### Perceived discrimination, transcultural identity and psychological adjustment

**Fig. 1:**
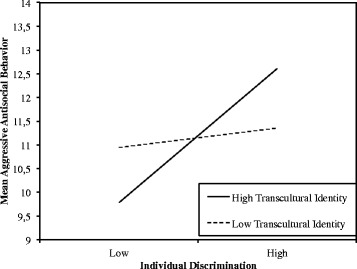
Interaction effect for Aggressive Antisocial Behavior When Perceiving Low vs. High Personal Discrimination, in Dependence of High vs. Low Levels of Transcultural Identity (Both Dichotomized by Median-Split)

Correlation analyses revealed that transcultural identity was not related to perceived discrimination (see Table 1). However, transcultural identity turned out to be a moderator variable for the relationship between perceived personal discrimination and psychological adjustment (see Table 3). Transcultural identity strengthened the relationship of perceived personal discrimination and poorer psychological adjustment. This moderator effect was present in the *overall psychological distress* measure and was explained in particular by the subscales of *externalizing symptoms*, i.e. aggressive antisocial behavior and anger control problems. Thus, among those participants who did not feel discriminated against, participants with a transcultural identity displayed better psychological adjustment when compared to participants with lower scores on the transcultural identity scale. On the contrary, among the participants who perceived higher rates of discrimination, those with a transcultural identity showed poorer psychological adjustment than their peers with lower scores on transcultural identity. Figure 1 illustrates the moderator effect of the variable transcultural identity, on the relationship between the independent variable *perceived discrimination* and the subscale *aggressive antisocial behavior* at the example of the dependent variable *psychological adjustment*.

**Table 3:** Moderator regression analyses predicting outcomes of psychological adjustment with perceived personal discrimination and transcultural identity

Outcome	Predictor	*B*	*SE B*	*ß*	*R* ^*2*^	Δ*R* ^*2*^
Aggressive antisocial behavior						
Step 1	Constant	11.20	.36		.33	
	Personal discr.	3.76	.81	.58**		
	Transcultural Identity	.28	.68	.05		
Step 2	Constant	11.25	.33		.45	.11**
	Personal discr.	5.24	.91	.81**		
	Transcultural Identity	.20	.62	.04		
	Personal d.xTranscult.	3.66	1.26	.40**		
Anger control problems						
Step 1	Constant	12.52	.42		.09	
	Personal discr.	2.44	.94	.37*		
	Transcultural Identity	.03	.79	.01		
Step 2	Constant	12.58	.39		.22	.14**
	Personal discr.	4.15	1.06	.63		
	Transcultural Identity	−.07	.73	−.01		
	Personal d.xTranscult.	4.22	1.48	.46**		
Overall psychological distress						
Step 1	Constant	50.39	.98		.35	
	Personal discr.	10.55	2.20	.59**		
	Transcultural Identity	.57	1.83	.04		
Step 2	Constant	50.51	.94		.42	.07*
	Personal discr.	13.83	2.55	.78**		
	Transcultural Identity	.39	1.75	.03		
	Personal d.xTranscult.	8.08	3.55	.33*		

*n* = 46. *p < .05 **p < .01

## Discussion

In order to gain further insights into potential risk and resiliency factors for the mental health of adolescents with a migratory background, the present study investigated perceived discrimination, psychological adjustment, as well as transcultural identity and examined the interplay of these factors. The results revealed that about two thirds of adolescents with a Turkish migratory background experienced strong or very strong discrimination in at least one of the areas of public life under investigation. This rate is higher than that found in a study of the German Federal Anti-Discrimination Agency (2012). In contrast to the present investigation, that study included immigrants of different age and origin. The higher rate in the present study is likely due to the selection of the sample, because persons with a Turkish migratory background, as well as younger people, are particularly affected by experiences of discrimination (German Federal Anti-Discrimination Agency 2012).

The present study distinguished between perceived group discrimination, i.e., the perception of discrimination for one’s group as a whole, and perceived personal discrimination, i.e., experiences of discrimination that happened to oneself personally. Results showed that respondents perceived more group discrimination than personal discrimination. This corresponds to the personal/group discrimination discrepancy that was also reported in previous studies (e.g., Taylor et al. 1990). As an explanation for the phenomenon, it has been discussed that personal discrimination might be denied for reasons of self-protection (Crosby 1984).

### Psychological adjustment of adolescents with a Turkish migratory background

We found that adolescents with a migratory background did not exhibit a poorer overall psychological adjustment when compared to participants without a migratory background. This is in line with recent studies (Haffner et al. 2006; Vollebergh et al. 2005; von Lersner et al., unpublished observations). Nevertheless, the results indicated that immigrant adolescents were more likely to report depressive symptoms. Interestingly, this difference disappeared when accounting for social status. This was also shown in a study by Murad et al. (2003) suggesting that socioeconomic measures, such as employment status and educational level of the parents, contribute to the explanation of differences in problem behavior between Turkish immigrant and native Dutch adolescents. Moreover, on average, immigrant families in Germany have a lower social status than families without a migratory background (ConsortiumEducation Report 2012). As lower social status is associated with a poorer mental health (Bøe et al. 2012; Ravens-Sieberer et al. 2008), conclusions about differences between adolescents with and without a migratory background should not be drawn without taking socioeconomic factors into account.

### Perceived discrimination and psychological adjustment

In the current study, perceived discrimination was clearly associated with poorer psychological adjustment. Accordingly, adolescents who perceived personal discrimination or group discrimination showed higher levels of depressive symptoms, aggressive antisocial behavior, anger control problems, overall psychological distress and somatic symptoms. This is in line with previous findings (Brody et al. 2006; Galliher et al. 2011; Lee & Ahn 2012). Even after controlling for demographic variables, the effect of perceived personal discrimination on the outcomes of psychological adjustment remained—except for depressive symptoms. Furthermore, diary studies show that anger is the most frequent affective response when perceiving discrimination (Swim et al. 2003). This is congruent to our finding that perceived discrimination was linked to externalizing symptoms. In addition, it should be noted that—when controlling for demographic variables—outcomes of psychological adjustment were predicted by perceived personal discrimination but not by perceived group discrimination (except for somatic symptoms that were also predicted by perceived group discrimination). This indicates that especially personally experienced discrimination plays a role for psychological adjustment. This finding can be important for further research when choosing instruments for the assessment of perceived discrimination and the interpretation of its effects.

### Perceived discrimination, transcultural identity and psychological adjustment

The current study introduced transcultural identity as a new operationalization of cultural identity based on the concept of transculturality (Welsch 1995). Transcultural identity is characterized by the dissolution of defined cultural boundaries and shaped by the incorporation of the ancestry culture, the local culture, and the global culture into one’s identity (Welsch 2010). The heterogeneity that results appears in different domains of everyday life like music, food, series and films on television, sports clubs, language, religion, annual festivals or clothing style. The current investigation explored whether perceived discrimination was associated with transcultural identity.

As a core finding of the study, transcultural identity moderated the relationship between perceived personal discrimination and psychological adjustment. In particular, this was true for aggressive antisocial behavior, anger control problems, and for overall psychological distress. Thus, compared to adolescents with a lower level of transcultural identity, adolescents with a higher level of transcultural identity had a better psychological adjustment, as long as they did not feel discriminated against. This finding is congruent with the idea that transcultural identity involves considerable benefits for personality, such as the ability to integrate elements of different cultures successfully into one’s identity (Welsch 2010). However, in the present study, adolescents with a transcultural identity displayed poorer psychological adjustment when they felt discriminated against. If transcultural identity is considered a resource, why is experiencing discrimination more detrimental for adolescents with a transcultural identity? There are several explanations for the strengthening effect of transcultural identity on the link of perceived discrimination and psychological adjustment.

First, it is useful to consider this finding in comparison to other concepts of identity such as ethnic identity. Ethnic identity is characterized by a feeling of belonging to the society of one’s origin (Phinney 1990) and, in contrast to transcultural identity, it buffers negative effects of perceived discrimination (Galliher et al. 2011; Mossakowski 2003; Vera et al. 2011; Wong et al. 2003). Thus, the feeling of belonging to an ingroup can serve as a source of support. Although transcultural persons also identify with the society of their origin, feelings of belonging are spread over different ingroups. Thus, our findings suggest that feelings of belonging then might be diffuse (Erikson 1963) and may not serve as a reliable buffer when perceiving discrimination.

Second, as transcultural identity involves the dissolution of defined cultural boundaries, transcultural persons also identify with groups other than that of their family’s origin. Consequently, there is an increased likelihood that transcultural persons experience discrimination from someone who belongs to a group they identify with. In this case, the experience of discrimination might re-establish seemingly overcome boundaries and provoke feelings of exclusion. As suggested by our findings this can call into question one’s identity and cause negative feelings.

Third, it needs to be taken into account that a certain set of additional attitudes and personality traits might be related to transcultural identity. For instance, transcultural identity could be associated with the attitude of being tolerant towards people who are different from oneself. Experiencing discrimination clearly stands in opposition to this attitude, and is prone to provoke anger and aggression. Accordingly, it is plausible that transcultural identity comes attached to openness to experience or to status-justifying beliefs. For example, status-justifying beliefs include the belief in a just world (Lerner 1980) and that status hierarchy is permeable (Sidanius & Pratto 1999). Major et al. (2007) theorized that perceiving discrimination threatens the world-view of individuals who endorse status-justifying beliefs (Major et al. 2007; Nelson 2009). Several psychological theories suggest that people experience a threat when their beliefs are challenged (Nelson 2009; Solomon et al. 1991). Accordingly, in further studies it will be of importance to investigate different additional attitudes or traits that might be related to transcultural identity.

In the present dataset, a main effect of transcultural identity on perceived discrimination was not found. Thus, persons with a transcultural identity did not systematically perceive less or more discrimination. This differs from previous findings on bicultural identity and ethnic identity: Benet-Martinez and Haritatos (2005) found that bicultural identity was associated with less perception of discrimination, and Operario and Fiske (2001) suggested that ethnic identity comes along with stronger perceptions of discrimination. However, Sellers et al. (2006) found that perceived discrimination was only associated with specific aspects of ethnic identity, such as *public regard* (e.g. “Others respect Black people”).

### Limitations and directions for further research

A few limitations should be noted when considering the results of the present study. First, the sample size was relatively small. Given the exploratory nature of this study, the generalizability of the present findings and conclusions could benefit from converging evidence in future studies with larger sample sizes.

Moreover, the findings of the present study do not allow causal inferences: due to the non-experimental design of the study, it is not clear if poorer psychological adjustment is really the consequence of perceived discrimination—it is also possible that adolescents with higher psychological distress are more susceptible to perceive or experience discrimination. Similarly, it could be that transcultural persons with mental health problems are particularly sensitive to perceiving discrimination. However, there are several longitudinal studies suggesting that discriminatory experiences precede mental health problems in adolescents (Brody et al. 2006; Galliher et al. 2011; Kim et al. 2011).

Another limitation concerns the assessment of the social status. Due to reasons of data protection, the research group did not have the permission to ask the participants directly about their social status. Therefore, social status was investigated approximately by asking the participants about the exemption from co-payments for school books. However, this remains an indirect measure of social status. In addition, this variable contains several missing values, such that the findings related to social status must be interpreted cautiously. Furthermore, perceived discrimination refers to the subjective point of view of the respondents, and could be confounded with certain personality traits. For instance, it is plausible that persons who are highly sensitive for rejection systematically perceive more discrimination than others (Major et al. 2003). Future investigations should accordingly control for additional personality traits, such as rejection sensitivity, when studying perceived discrimination. Moreover, it would be interesting to investigate whether adolescents with a low transcultural identity identify more strongly with another particular identity, such as ethnic identity.

Additionally, it needs to be taken into account that the participants of this study were aged between 14 and 17 years. A proportion of them may not have fully developed their cultural identity yet. Therefore, it would be interesting to conduct this study with adult participants, whose cultural identity should be more established. Finally, it would be interesting to investigate if the moderator effect generalizes to other regions and groups other than adolescents with a Turkish migratory background.

## Conclusions

The present study contributes to a better understanding of the psychological adjustment of adolescents with a migratory background. We investigated its relation to perceived discrimination and transcultural identity—a new operationalization of cultural identity. Although further studies are needed to clarify the exact nature of transcultural identity, the findings of the present study contain important theoretical and practical implications.

We show that perceived discrimination is associated with poorer psychological adjustment in adolescents with a migratory background. Moreover, the current study provides evidence that transcultural identity moderates this relationship. Within the group of adolescents who do not feel discriminated against, those with a transcultural identity display a better psychological adjustment. This is congruent with the idea that transcultural identity involves considerable benefits for personality. However, when regarding adolescents who report higher levels of discrimination, the persons with a transcultural identity show a poorer psychological adjustment. Thus, when comparing the well-being of adolescents with lower and higher levels of transcultural identity, the well-being of the persons with higher levels of transcultural identity is more dependent upon experiences of discrimination. Due to evolving changes and movements in our contemporary world, transcultural identity is a form of cultural identity that we are likely to encounter more frequently. Moreover, it is a highly desirable form of cultural identity that should be encouraged. Hence, it is essential to know about this specific vulnerability vis-à-vis perceived discrimination in order to prevent or treat mental health problems adequately. However, supporting adolescents to cope with experiences of discrimination is not enough. It is also important to keep on raising awareness about the detrimental effects of obvious, as well as subtle ethnic discrimination, and about the relevance of appreciating diversity in society.

## Figures and Tables

**Fig. 2:**
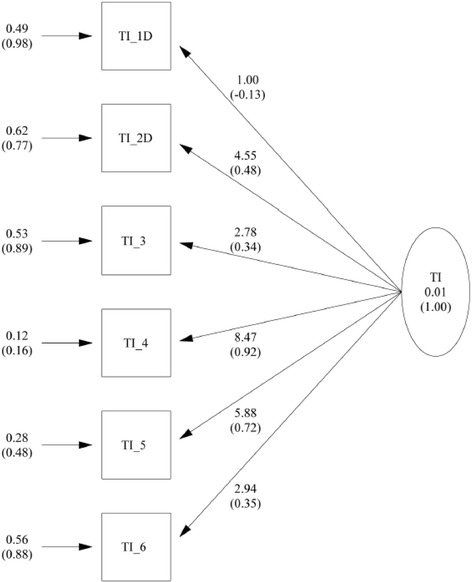
Factorial Structure of Transcultural Identity in Adolescents Without a Migratory Background (The figure was derived from Günther, K. (2012). *Transcultural Identity in Adolescents With and Without Migration Background*. Unpublished Thesis, Humboldt-Universität zu Berlin, Berlin, Germany.)

**Fig. 3:**
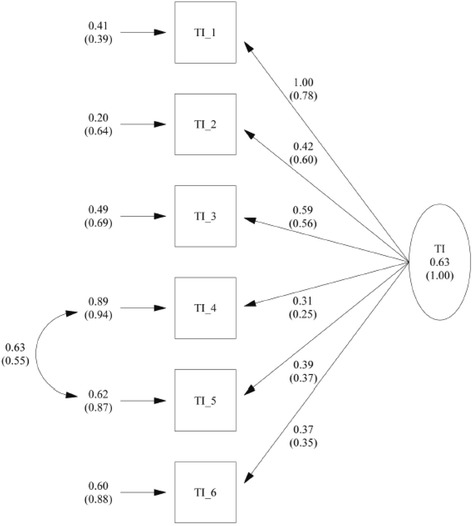
Factorial Structure of Transcultural Identity in Adolescents with a Migratory Background (The figure was derived from: Günther, K. *Transcultural Identity in Adolescents With and Without Migration Background.* Unpublished thesis (2012))

## Appendix

### Evaluation of the transcultural identity questionnaire

We conducted an item analysis of the Transcultural Identity Questionnaire by regarding the two groups of participants with and without a migratory background individually because items one and two differed between the groups. For adolescents with a migratory background, item difficulties were medium (.22 < *p* < .37) except item TI_2, which tended to be difficult (*p* = .07). Corrected inter-item-correlations were also in a medium range (.31 < *r* < .51). In the group of adolescents without a migratory background, item difficulty and corrected inter-item-correlations were also in a medium range (.22 < *p* < .51; .34 < *r* < .64) except item TI_1D (*p* = .09; *r* = .2) and TI_6 (*p* = .18; *r* = .2).

In order to validate the structure of the Transcultural Identity Questionnaire we tested the independence of the concepts of bi- and transcultural identity using a structural equation model (SEM). For adolescents without a migratory background the analysis showed a unidimensional model with all six items loading on the latent construct transcultural identity (see Fig. 2). The resulting fit for this model was: χ^2^(9) = 8.25 (*p* = .51), CFI = 1, RMSEA < .001 (90 % CI: <. 001–.158), SRMR = .07. Construct reliability was Ω = .88. All loadings exceeded a ≥ .13 and were significant. Overall the model fit was good.

For adolescents with a migratory background the analysis also showed a unidimensional model with all six items loading on the latent construct transcultural Identity. Here, the model was modified once as indicated by the modification indices, so that error variables of item 4 and 5 were correlated (see Fig. 3). The resulting fit for this model was: *χ*^2^ (8) = 10.96 (*p* = .2), CFI = .94, RMSEA = .09 (90% CI: < .001–.208), SRMR = .08. Construct reliability was Ω = .75. The minimum loading was a = .25 and all but one (TI_4) were significant. The model fit was good.

In both groups, items were associated with the latent variable – indicating that similar constructs were measured in the both groups.

## Abbreviations

BII: Bicultural Identity Integration
SPS-J: Screening of psychological disorders in adolescence
